# Incorporating spatial diffusion into models of bursty stochastic transcription

**DOI:** 10.1098/rsif.2024.0739

**Published:** 2025-04-09

**Authors:** Christopher E. Miles

**Affiliations:** ^1^ Department of Mathematics, Center for Complex Biological Systems, University of California, Irvine, CA, USA

**Keywords:** gene expression, stochastic processes, spatial transcriptomics, modelling and inference

## Abstract

The dynamics of gene expression are stochastic and spatial at the molecular scale, with messenger RNA (mRNA) transcribed at specific nuclear locations and then transported to the nuclear boundary for export. Consequently, the spatial distributions of these molecules encode their underlying dynamics. While mechanistic models for molecular counts have revealed numerous insights into gene expression, they have largely neglected now-available subcellular spatial resolution down to individual molecules. Owing to the technical challenges inherent in spatial stochastic processes, tools for studying these subcellular spatial patterns are still limited. Here, we introduce a spatial stochastic model of nuclear mRNA with two-state (telegraph) transcriptional dynamics. Observations of the model can be concisely described as following a spatial Cox process driven by a stochastically switching partial differential equation. We derive analytical solutions for spatial and demographic moments and validate them with simulations. We show that the distribution of mRNA counts can be accurately approximated by a Poisson-beta distribution with tractable parameters, even with complex spatial dynamics. This observation allows for efficient parameter inference demonstrated on synthetic data. Altogether, our work adds progress towards a new frontier of subcellular spatial resolution in inferring the dynamics of gene expression from static snapshot data.

## 1. Introduction

Gene expression at the molecular scale is stochastic [1,2]. The consequences of this variability span development and disease [3–5]. Over the past decades, a vast body of research has evolved on constructing and analysing increasingly intricate biophysical models to disentangle the sources and functions of gene expression stochasticity [6–13]. More recently, these mechanistic models have also seen uptake and success in revealing insights from messenger RNA (mRNA) count distributions from imaging and sequencing transcriptomics technologies [14–21].

Intertwined with stochasticity, gene expression is also an inherently spatial process at the subcellular scale [22]. After transcription at distinct locations within the highly structured nucleus, mRNA must be transported through the nuclear interior to be exported through nuclear pores [23]. This spatial transport and export of mRNA into the cytoplasm is followed by translation into proteins and serves as a fundamental regulation of gene expression [24–27]. Imaging technologies now give access to observing these subcellular spatial processes at unprecedented resolution. For instance, single-molecule fluorescence *in situ* hybridization (smFISH) [28,29] provides spatial locations of individual mRNA molecules within nuclei and cytoplasm for multiple genes [30,31]. State-of-the-art analysis of this subcellular spatial data is largely phenomenological [32–34] and challenging to associate with biophysical mechanisms. Models that do mechanistically account for nuclear export largely do so by treating the nucleus as a homogeneous compartment [35–40] and do not readily incorporate fine-grained spatial features that influence mRNA dynamics. For instance, nuclear geometries [41] and transcription site locations [42] vary per cell even for the same gene, both of which shape the timescale of mRNA export. Perhaps even more importantly, spatial locations of mRNA encode the underlying dynamics of their production and degradation, information that is discarded by considering only counts. In summary, important subcellular spatial details are readily available from imaging, but the ability to incorporate them into current mechanistic modelling machinery is lacking. Motivated by this gap between data and theory, this work pursues the advancement of stochastic models of gene expression to the next frontier of subcellular spatial resolution.

The slow progress towards faithful subcellular spatial models of gene expression is an outcome of the immense challenges involved. Beyond the staggering complexities of the spatial organization of the nucleus, even simple spatial stochastic models have considerable technical obstacles facing their uptake [43]. For non-spatial models, the pursuit is a scalar stochastic quantity described by a distribution. The addition of space increases the complexity dramatically with both stochastic numbers and spatial locations of interest. From a computational perspective, this hurdle cannot be overstated. For instance, consider a two-dimensional smFISH image discretized into N×N pixels. There is a temptation to use any of the zoo of techniques that have enjoyed success for non-spatial models, including generating functions [44], finite-state projections [16,45], or neural networks [46,47]. However, one would seemingly need to perform these sometimes already costly or complex calculations for all pixels in the image, each with a distribution for counts. Even for a modest N∼100, solving for $N^{2}$ distributions for a single image becomes computationally prohibitive. Therefore, the analysis of spatial stochastic models for gene expression must be carefully considered to ensure that it remains computationally tractable enough to be associated with data.

To address the computational challenges of spatial stochasticity, we use a spatial point process formulation of the problem [48]. Specifically, we build upon our recent work [49] that investigates inference of the dynamics in a model of nuclear mRNA that undergoes spatial stochastic birth, death, and diffusion. The resulting description is a spatial Poisson process with an intensity that is the solution of a deterministic partial differential equation (PDE). This description allows for the rapid evaluation of a mechanistic likelihood that encodes both the stochastic number and positions of the particles simultaneously, including the important complication of heterogeneities [15,41]. With knowledge of the diffusion coefficient (measurable through live-cell tracking [50]), both the birth rate λ and death rate γ can be recovered from steady-state snapshot spatial data. This is in contrast to the non-spatial birth-death process, where only the ratio λ/γ can be recovered, highlighting the value of spatial information even for purely demographic inquiries.

The mathematics of our previous work hinges on a Poissonian birth process corresponding to a constitutive gene. The constitutive model neglects the bursty behaviour of transcription [51] and consequently fails to reconcile the dispersion (variance relative to the mean) of RNA counts seen in real data [52–54]. In this work, we extend the point process framework to a more realistic telegraph model [55] of transcriptional activity that stochastically switches on and off. The introduction of particle correlations creates a technical obstacle unaddressed with the previous work’s machinery. Here, we follow [48] and use the spatial Poisson representation of the process [56,57] that yields a stochastic partial differential equation (SPDE) for the intensity of a Poisson process, making it a Cox process. Motivated by the success of the Poisson representation in deriving valuable analytical results for non-spatial models [58–61], the focus of this work is interrogating exact and approximate analytical results on this SPDE-driven point process model for spatial bursty gene expression.

The outline of the article is as follows. We first introduce the formulation of the spatial stochastic model of nuclear mRNA with stochastically switching transcription. Using the spatial Poisson representation, we show that observations of the model follow a Cox process whose intensity is described by a stochastically switching PDE. For a one-dimensional model, we analytically compute spatial and demographic moments for the point process, including verification by comparison with stochastic simulations. The value of these moments is limited, but we identify that the full distribution of counts can be well approximated by a Poisson-beta distribution with analytically tractable parameters that encode the spatial effects. This distributional approximation is shown to extend to more realistic variations of the model, including a semi-reflecting boundary that approximates pores on the nuclear surface, and more realistic cell shapes with heterogeneous interiors. The results culminate into a proof-of-concept demonstration of inference on synthetic data with heterogeneous cell shapes and gene locations. Altogether, the work paves a clearer path towards mechanistic model-based inference of stochastic gene expression that faithfully incorporates subcellular spatial dynamics.

## 2. Model

**Figure 1.**
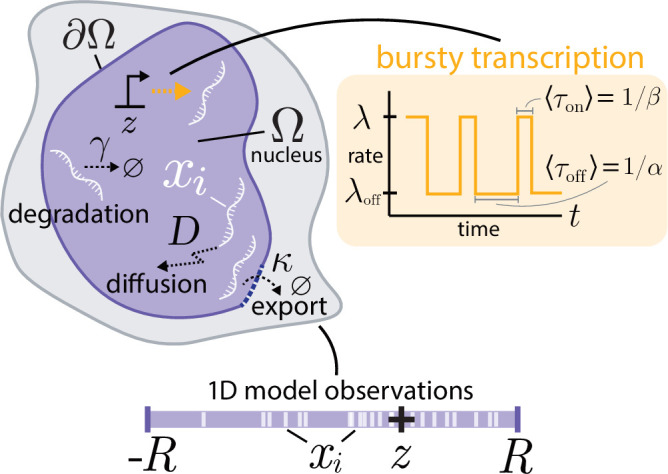
Schematic of the nuclear mRNA model. Transcription occurs at a spatial location z at a rate that stochastically switches between λ and $\lambda_{\mathrm{off}}$ at rates α,β. After transcription, mRNA diffuse with diffusivity D until degradation at rate γ or export at the boundary, controlled by parameter κ. Throughout the article, we primarily consider a one-dimensional spatial model with Ω=[−R,R].

The model considered here is a spatial stochastic description of individual molecules of mRNA in the nucleus of a cell, shown schematically in figure 1. The nuclear geometry is encoded in a domain Ω with boundary ∂Ω. The mRNA molecules are created through transcription at a fixed gene spatial location z∈Ω, at a stochastically varying rate based on a discrete promoter state. The transcription rate follows a dichotomous noise process [62], switching between an ‘on’ rate λ and an ‘off’ rate $\lambda_{\mathrm{off}}$, at rates α,β, respectively. Once transcribed, mRNA diffuses in the nuclear geometry with diffusivity D until they face two possible outcomes: degradation at rate γ or exportation at the nuclear boundary. Nuclear export rates are encoded in a parameter κ discussed in further detail later. Heuristically, κ=0 corresponds to no exportation (a purely reflecting boundary) and κ=∞ describes the instantaneous export of RNA at the nuclear boundary (purely absorbing). The model observations correspond to the spatial positions of the RNA $x_{1},\ldots ,x_{n}$ within the nucleus, where both positions and numbers are stochastically evolving. Throughout, only steady-state dynamics t→∞ are considered, acknowledging the neglect of transient effects from cell-cycle dynamics [13,39,63]. For illustrative purposes throughout the article, the domain is considered to be one spatial dimension with Ω=[−R,R], with R crudely interpreted as the nuclear radius.

The rest of the manuscript pursues statistical descriptions of observations from this model. We reiterate the challenges for emphasis. Both the number and locations of the molecules are stochastic. Moreover, the temporally correlated nature of the birth process induces correlations between the molecules, so they may not be considered independently. These challenges are alleviated by the discovery that the model enjoys a straightforward spatial Poisson representation [48,56,57,64]. With more details shown in appendix A, we show that the particle locations follow the spatial Poisson process:


(2.1) $$\begin{array}{lcr} & x_{1},\ldots ,x_{n}∼\mathrm{Poiss}(u(x)), & \end{array}$$

where the intensity u(x) corresponds to the steady-state distributional solution u(x,t) of the SPDE:


(2.2) $$\begin{array}{lcr} & \partial_{t}u=D\nabla^{2}u-\gamma u+\Lambda (t)\delta (x-z). & \end{array}$$

In equation (2.2), δ(x) is the Dirac delta function and Λ(t) is the continuous-time (asymmetric) dichotomous noise process, switching between values $\left\{\lambda_{\mathrm{off}},\lambda \right\}$, at rates {α,β}, summarized by:


(2.3) $$\begin{array}{lcr} & \Lambda (t):\;\lambda_{\mathrm{off}}\underset{\alpha }{\overset{\beta }{⇆}}\lambda . & \end{array}$$

The SPDE equation (2.2) can be written as a stochastically switching PDE [65]:


(2.4) $$\begin{array}{r} \begin{array}{rl} \partial_{t}u & =D\nabla^{2}u-\gamma u+\lambda_{off}\delta (x-z)\\ & \beta ↑↓\alpha \\ \partial_{t}u & =D\nabla^{2}u-\gamma u+\lambda \delta (x-z). \end{array} \end{array}$$

The stochastic nature of the intensity u(x,t) in the Poisson process (equation (2.1)) makes the observations $x_{1},\ldots ,x_{n}$ a spatial Cox process, a doubly stochastic Poisson process. We defer a discussion of boundary conditions for now.

It is worthwhile to note that the contrast to the purely Poissonian birth (constitutive gene) case. If transcripts are produced at a constant rate λ, the Poisson process (equation (2.1)) remains but now with a purely deterministic intensity:


$$\begin{array}{c} \partial_{t}u=D\nabla^{2}u-\gamma u+\lambda \delta (x-z). \end{array}$$

In this Poissonian birth scenario, the particles are entirely independent from each other. Therefore, the stochastic intensity underlying the point process can be attributed to arising from the correlated nature of the particles.

The Poisson representation provides clarity in encoding the locations and stochastic counts concisely. However, the resulting Poisson point process with an intensity driven by an SPDE is not immediately illuminating. This leads us to pursue calculating emergent properties from this formulation.

## 3. Model analysis

### 3.1. Mean behaviour

Our starting point for analysis of the model is in computing moments for both spatial positions and molecular counts. The mean behaviour is straightforward to compute. Taking averages of equation (2.2) with respect to the realizations of Λ(t) gives the deterministic PDE for the average ⟨u(x,t)⟩:


(3.1) $$\begin{array}{lcr} & \partial_{t}\langle u\rangle =D\nabla^{2}\langle u\rangle -\gamma \langle u\rangle +\frac{\alpha \lambda +\beta \lambda_{\mathrm{off}}}{\alpha +\beta }\delta (x-z). & \end{array}$$

The mean of the counts for the Cox process (equation (2.1)) is the integrated mean intensity [66]:


(3.2) $$\begin{array}{lcr} & \langle n(t)\rangle =\int_{\Omega }\langle u(x,t)\rangle dx. & \end{array}$$

For ease of digesting the resulting formulae, we take $\lambda_{\mathrm{off}}=0$, κ=∞, and Ω=[−R,R] for now. In the steady-state limit t→∞ equation (3.1) becomes the boundary value problem:


(3.3) $$\begin{array}{lcr} & \begin{array}{rl} 0 & =D\partial_{xx}\langle u\rangle -\gamma \langle u\rangle +\frac{\alpha \lambda }{\alpha +\beta }\delta (x-z),\\ & \;\langle u(-R)\rangle =0,\;\;\langle u(R)\rangle =0. \end{array} & \end{array}$$

Calling ρ=α/(α+β), the fraction of time the transcription activity is on, the solution of equation (3.3) is:


(3.4) $$\begin{array}{rl} \left\langle u(x)\right\rangle & =\;\frac{\lambda \rho }{2\sqrt{\gamma D}}\text{csch}\left(2R\sqrt{\gamma /D}\right)[\cosh\left(\sqrt{\gamma /D}(2R-|x-z|)\right)-\cosh\left(\sqrt{\gamma /D}(x+z)\right)]. \end{array}$$

By equation (3.2), the total average number of molecules can then be computed by integrating equation (3.4):


(3.5) $$\begin{array}{lcr} & \begin{array}{rl} \left\langle n\right\rangle & =\frac{\lambda \rho }{\gamma }\left[1-\cosh\left(z\sqrt{\gamma /D}\right)\;\mathrm{sech}\left(R\sqrt{\gamma /D}\right)\right].\\ & :=\frac{\lambda \rho }{\gamma }S. \end{array} & \end{array}$$

The resulting mean (equation (3.5)) deserves interpretation. The dimensionless scale factor S∈[0,1] reflects the level of export out of the boundaries. As S→1, the purely non-spatial mean is recovered and space plays no role in the molecular counts. Otherwise, S decreases with any factor that increases the overall flux out of the boundary: faster diffusion, smaller domains or z closer to the boundary. S is an increasing function of γ, suggesting an interpretation of S as the probability a molecule is exported *before* it is degraded.

**Figure 2.**
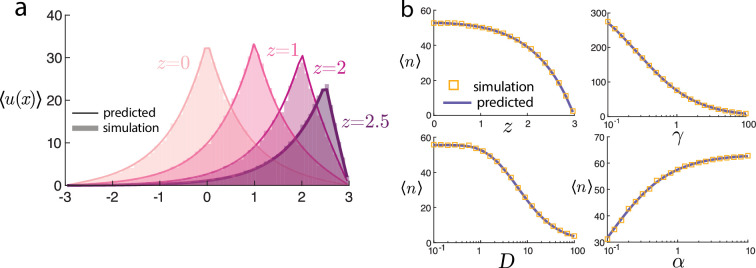
Mean steady-state behaviour of the model. (a) Mean positions ⟨u(x)⟩ from equation (3.4) varying source locations z. (b) Mean number of particles ⟨n⟩ from equation (3.5) for various parameter sweeps. Simulations and predicted values closely agree.

Verification of the predicted means (equations (3.4) and (3.5)) can be seen in figure 2. In panel (*a*), the mean spatial position is shown for varying gene site location z, and all other parameters are fixed. As the gene site shifts closer to the boundary, the overall intensity level decreases. This is further highlighted in panel (*b*), where the average total number of molecules goes to zero as the gene site approaches the boundary. The mean ⟨n⟩ also decreases with the degradation rate γ and diffusivity D, but increases with α. The predicted means and stochastic simulations show near-perfect agreement. Further details on the stochastic simulation can be found in appendix B.

### 3.2. Variance of the molecular counts

The mean behaviour of the telegraph model is effectively indistinguishable from Poissonian production with the lumped parameter λp as the effective production rate. We anticipate that higher order moments do not bear this equivalence.

The variance for the total number of particles of the Cox process (equation (2.1)) can be computed by:


(3.6) $$\begin{array}{lcr} & \mathrm{var}(n)=\langle n\rangle +\int_{\Omega }\mathrm{var}(u(x))dx. & \end{array}$$

For the constitutive case, the intensity is deterministic, and var⁡(u(x))=0 so that var⁡(n)=⟨n⟩ and a Poisson distribution is recovered. For the bursty process, u(x,t) evolves stochastically (equation (2.2)), so fluctuations in the intensity also manifest in contributing super-Poissonian variance to the molecular counts. The mean ⟨n⟩ was computed in the previous section, so the determination of the variance of the counts is left to determine the variance of the intensity.

Continuing with purely absorbing boundaries κ=∞ in one spatial dimension Ω=[−R,R], we compute $\int_{\Omega }\mathrm{var}(u)dx$ after a lengthy calculation. The result is a doubly infinite sum:


(3.7) $$\int_{\Omega }\mathrm{var}(u)dx=\psi \sum_{m=1}^{\infty }\sum_{n=1}^{\infty }\frac{\mu_{2m-1}+\mu_{2n-1}+2\omega }{\left(\mu_{2m-1}+\omega )(\mu_{2n-1}+\omega )(\mu_{2m-1}+\mu_{2n-1}\right)}\times \frac{16\sin\left(\frac{\pi (2m-1)(R+z)}{2R}\right)\sin\left(\frac{\pi (2n-1)(R+z)}{2R}\right)}{\pi^{2}(2m-1)(2n-1)},$$

where $\psi =\lambda^{2}\alpha \beta /(\alpha +\beta {)}^{2}$, ω=α+β, and $\mu_{m}:=-\gamma -\pi^{2}Dm^{2}/(4R^{2})$. The calculation largely follows [67,68] on the stochastic cable equation. The key observation is to note that the SPDE solution to equation (2.2) can formally be written as:


(3.8) $$\begin{array}{lcr} & u(x,t)=\int_{0}^{t}G(x,t-s)\Lambda (s)ds, & \end{array}$$

where G(x,t) is the appropriate Green’s function with a textbook series solution [69]. This Green’s function is combined with moments of dichotomous noise Λ(t) to yield this series solution for the integrated variance of the intensity. Further details of the calculation can be found in appendix C.

**Figure 3.**
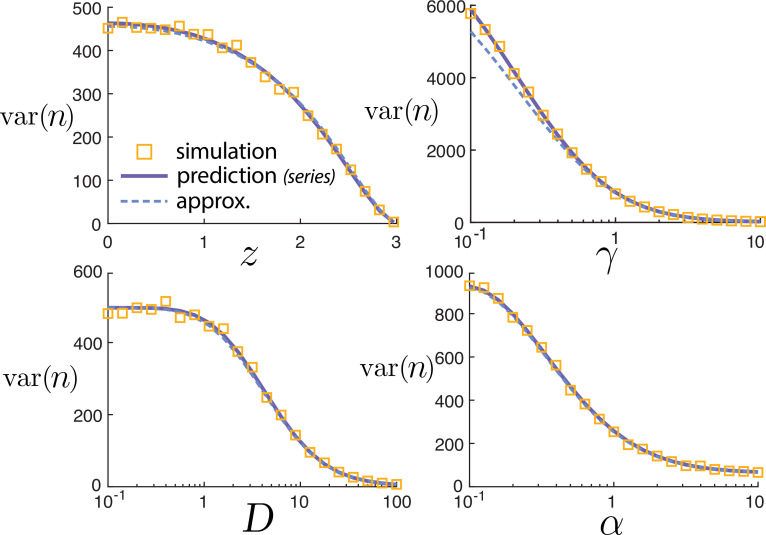
Variance of the number of molecules var⁡(n) for various parameter sweeps. Simulations and predicted values from the infinite series (dashed line) solution (3.7) closely agree. Moreover, the approximate scaling value from the Poisson-beta distribution (equation (3.10)) (solid line) is nearly (but not exactly) identical over the parameter ranges.

To the best of our knowledge and Mathematica’s abilities to simplify symbolic expressions, the infinite series (equation (3.7)) does not afford an elementary expression. It can be evaluated straightforwardly numerically. A comparison between the variance var⁡(n) predicted by the series solution can be seen in figure 3. The parameters D,γ,α, and z→R all yield a decrease in the variance of molecular counts. Furthermore, there is close agreement between the series prediction (equation (3.7)) and the stochastic simulations.

### 3.3. Poisson-beta distributional approximation

The difficulty in computing and digesting this series solution lends little hope to the direction of generalizing this machinery to more complex set‐ups. Moreover, these moments are computed over realizations of the process in the same domain. Since the cell shape and gene site vary with each observation, it is not clear how moments may be directly connected to data with these heterogeneities. Instead, we identify a simple approximate description that will turn out to be surprisingly useful and accurate.

We motivate the approximation by reminding the reader that the mean molecular counts in equation (3.5) is Sλα/(α+β), with the terms multiplying S interpreted as the non-spatial mean. One could arrive at this same answer by rescaling the parameters of the non-spatial process λ→Sλ/γ, α→Sα/γ, and β→Sβ/γ. The division of γ arises from the lack of identifiability of a single parameter (time scale) in steady state. The factor of S will be the basis of the approximation.

The non-spatial variance is [7,61]:


(3.9) $$\mathrm{var}(n{)}_{\text{non-spat}}=\langle n\rangle_{\text{non-spat}}+\frac{\alpha \beta \lambda^{2}}{\left(\alpha +\beta {)}^{2}\gamma (\alpha +\beta +\gamma \right)}.$$

If we follow the same line of reasoning and rescale parameters λ→Sλ/γ, α→Sα/γ and β→Sβ/γ to account for spatial effects, this suggests:


(3.10) $$\begin{array}{lcr} & \mathrm{var}(n)\approx \langle n\rangle +\frac{S^{2}\alpha \beta \lambda^{2}}{S(\alpha +\beta {)}^{2}\gamma (\alpha +\beta +\gamma )}. & \end{array}$$

Values for this expression are plotted alongside the series solution in figure 3. Although this predicted value for the variance is distinct from the series prediction, the values are nearly indistinguishable over all parameters considered.

The non-spatial variance (equation (3.9)) corresponds to a Poisson-beta distribution for the molecular counts, parameterized by three values $\tilde{\alpha }$, $\tilde{\beta }$, $\tilde{\lambda }$:


(3.11) $$\begin{array}{rl} p(n;\tilde{\alpha },\;\tilde{\beta },\tilde{\lambda }{)}_{PB} & =Poiss(n;\tilde{\lambda t})\wedge_{t}Beta(t;\;\tilde{\alpha },\tilde{\beta })=\int_{0}^{1}\frac{\tilde{\mu }t}{n!}e^{-\tilde{\mu }t}\frac{t^{\tilde{\alpha }-1}(1-t{)}^{\tilde{\beta }-1}}{B(\tilde{\alpha },\tilde{\beta })}dt=\frac{{\tilde{\mu }}^{n}}{n!}\frac{(\tilde{\alpha }{)}_{n}}{(\tilde{\alpha }+\tilde{\beta }{)}_{n}}{\;}_{1}F_{1}(\tilde{\alpha }+n,\tilde{\beta }+n;-\tilde{\lambda }), \end{array}$$

where $\wedge_{t}$ denotes the mixture distribution with respect to t, ${\;}_{1}F_{1}$ is the confluent hypergeometric function, and $\left(c{)}_{n}=c(c+1)\cdots (c+n-1\right)$ is the Pochhammer symbol.

**Figure 4.**
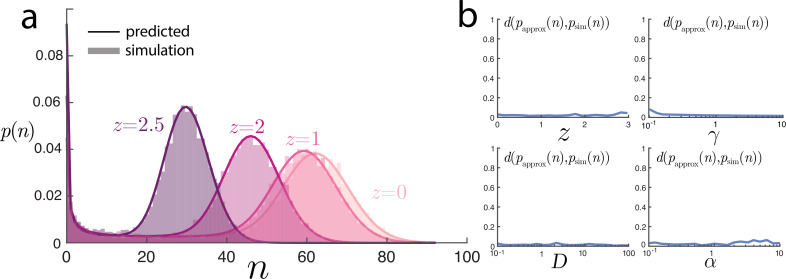
Comparison of the Poisson-beta distributional predictions with simulations. (a) For the same set‐ups shown in 2 a with varying z, the predicted full distribution of the number of molecules closely agrees with simulations. (b) Over the parameter sweeps in figures 2 and 3, the Jensen–Shannon distance (between 0 and 1) between the predicted Poisson-beta distribution of n and the values from simulations are consistently small, highlighting the broad applicability of the approximation.

The spatial variance approximation (equation (3.10)) is recovered with the choice of $\tilde{\lambda }=S\lambda /\gamma$, $\tilde{\alpha }=S\alpha /\gamma$, and $\tilde{\beta }=S\beta /\gamma$. Although this expression was obtained by heuristic scaling arguments of the moments, we can plot the full distribution with these parameter choices against the values from stochastic simulations. Figure 4 shows these comparisons with remarkable agreement. In figure 4a, we demonstrate the predicted distribution of n for the same set‐up as figure 2a and near-identical agreement is seen for all values of n.

One should be sceptical about the validity of this approximation. To investigate this, we computed the Jensen–Shannon divergence $d_{JS}(p_{1},p_{2}):=[d_{KL}(p_{1}|\frac{p_{1}+p_{2}}{2})+d_{KL}(p_{2}|\frac{p_{1}+p_{2}}{2})]/2$ between the predicted Poisson-beta distribution and the empirical distribution from simulations. This divergence takes values between 0 and 1, and across all parameter ranges tested shown in figure 4, the values were on the order of ≈0.01, suggesting remarkable agreement.

### 3.4. Generalizing to semi-absorbing boundaries

The Poisson-Beta distribution provides an accurate prediction for the full distribution of molecular counts for purely absorbing boundaries and only requires a single deterministic PDE solution. To demonstrate this approach’s surprisingly broader applicability, we first extend the model to account for more realistic nuclear export. Consider a Robin boundary condition for the SPDE (2.2):


(3.12) $$\begin{array}{lcr} & D\nabla u(x,t)\cdot n(x){|}_{x\in \partial_{\Omega }}=\kappa u(x,t){|}_{x\in \partial_{\Omega }}, & \end{array}$$

where n is the outward normal vector to the boundary ∂Ω. Such a boundary condition can arise from homogenizing the surface of the nucleus with absorbing pores [70,71]. We interpret κ as controlling the kinetics of nuclear export [27]. With these boundary conditions, the PDE for the steady-state mean intensity ⟨u(x)⟩ (x)⟩ (equation (3.3)) now becomes:


(3.13) $$\begin{array}{rl} 0 & =D\partial_{xx}\langle u\rangle -\gamma \langle u\rangle +\frac{\alpha \lambda }{\alpha +\beta }\delta (x-z),\\ & \;D\langle \partial_{x}u(-R)\rangle =\kappa \langle u(-R)\rangle ,\;-D\langle \partial_{x}u(R)\rangle =\kappa \langle u(R)\rangle . \end{array}$$

This can again be solved directly, and by (equation (3.2)) the mean number of molecules can be computed from the integral over the mean intensity, yielding a similar result to the κ=∞ case (equation (3.5)):


(3.14) $$\langle n\rangle =\int_{-R}^{R}\langle u(x)\rangle dx=\frac{\lambda \rho }{\gamma }S_{\kappa },$$

where $S_{\kappa }$ is a slightly unwieldy but straightforward to compute expression


(3.15) $$\begin{array}{lcr} & S_{\kappa }=\frac{\sqrt{\gamma D}-\left(\sqrt{\gamma D}+\kappa \right)e^{2R\sqrt{\frac{\gamma }{D}}}+\kappa e^{\sqrt{\frac{\gamma }{D}}(R-z)}+\kappa e^{\sqrt{\frac{\gamma }{D}}(R+z)}-\kappa }{-\sqrt{\gamma D}+\left(\sqrt{\gamma D}+\kappa \right)e^{2R\sqrt{\frac{\gamma }{D}}}+\kappa }. & \end{array}$$

**Figure 5.**
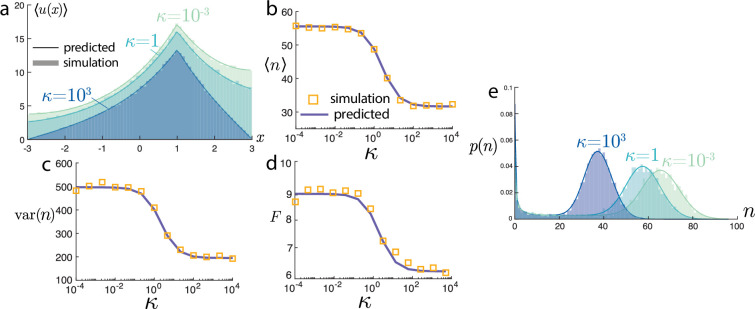
Influence of the export rate parameter κ. (a) Mean positions ⟨u(x)⟩ for varying levels of κ from (3.13). (b) Mean number from (3.14), (c) variance, and (d) Fano factor F=var(n)/⟨n⟩ of molecular counts all decrease with κ. (e) The Poisson-beta predicted distribution shows close agreement with stochastic simulations for various κ.

This scaling factor has the same interpretation: $S_{\kappa }\in \left[0,1\right]$ and can be understood as the probability of export before degradation. As $\kappa \to 0,S_{\kappa }\to 0$, since no export occurs. On the other hand, as κ→∞, $S_{\kappa }\to S$, the fully absorbing limit defined in equation (3.5). In figure 5*a*,*b*, we show close agreement between stochastic simulations and the predicted mean behaviour. As κ gets smaller, the number of molecules increases, and the intensity profile flattens out. In stochastic simulations, κ is interpreted in the partially reflected sense [72], where larger κ encodes a higher probability of exit. Further simulation details can be found in appendix B.

We now consider the same parameter scaling for a Poisson-beta distribution, $\tilde{\lambda }=S_{\kappa }\lambda /\gamma$, $\tilde{\alpha }=S_{\kappa }\alpha /\gamma$, and $\tilde{\beta }=S_{\kappa }\beta /\gamma$. In figure 5*c*,*d*, we show the predicted variance and Fano factor F=var(n)/⟨n⟩. As κ increases, both the variance and Fano factor decrease, in agreement with experimental findings of [25] that show slowing down export (lower κ) leads to an increase in the nuclear mRNA Fano factor for several genes. Moreover, over this same range of κ, the Poisson-Beta prediction provides a remarkably accurate prediction for the full distribution of molecular counts, shown in figure 5*e*. This predicted distribution required only the computation of the deterministic PDE for the mean behaviour (equation (3.14)). By contrast, the full variance calculation would have required tedious computations, ultimately likely to also result in an unwieldy infinite series solution. Thus, the finite κ scenario highlights the use of the Poisson-beta approximation.

### 3.5. Fano factor interpretation of spatial effects

**Figure 6.**
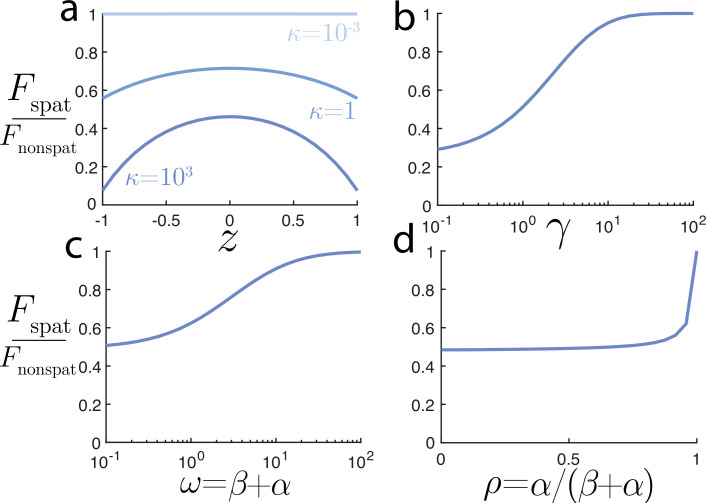
Comparison of the Fano factors for spatial and non-spatial models. (a) Source locations with the largest κ and closest to the boundary have the largest deviation between spatial and non-spatial models. (b) The degradation rate γ and (c) transcriptional switching timescale ω both increase the agreement between the spatial and non-spatial Fano factors. (d) The proportion of time in the ‘on’ state, p has a relatively weak influence on the Fano factor ratio, except for nearly constitutive p≈1.


We are now equipped with the ability to study the nuclear mRNA model’s statistical behaviour for various parameters and model variants. Before continuing to generalize the model, we take a brief interlude to highlight the value of an explicit spatial model in interpreting molecular counts of nuclear mRNA. In figure 6, we compare the Fano factor for the explicit spatial model:

(3.16) $$F_{spat}=\frac{\mathrm{var}(n)}{\left\langle n\right\rangle }=1+\frac{\lambda \beta S}{\left(\alpha +\beta )(\gamma +S(\alpha +\beta )\right)},$$

to the nonspatial variant which neglects nuclear export:


(3.17) $$F_{non-spat}=1+\frac{\lambda \beta }{\left(\alpha +\beta )(\gamma +(\alpha +\beta )\right)}.$$

In figure 6a, we see that the largest deviation between the Fano factors of the spatial and non-spatial models occurs when exportation occurs most frequently: a gene site near the boundary (z≈R) and fast export (κ large). As the gene site moves to the interior of the nucleus or exportation slows, the two models more closely agree in their prediction. Notably, the Fano factor for the spatial model is always smaller than that of the non-spatial. Taken together, this reduction can be straightforwardly understood by noting that export removes molecules from the count and therefore reduces the overall fluctuations. This point is supported in figure 6b, where larger values of the degradation rate γ are shown to lead to the smallest deviation between the spatial and non-spatial models. This is expected, as larger γ means the outcome of transcripts is dominated by degradation, and export plays less of a role in their dynamics. The interplay between export and the transcriptional state is less easy to predict. In figure 6*c*,*d*, we see that slow transcriptional switching dynamics (small ω=α+β) leads to the biggest deviation between spatial and non-spatial variants. Moreover, the deviation is relatively robust to the fraction of time the transcription state is on, p=α/(α+β), except when p≈1 and production approaches a constitutive Poissonian process. Although these deviations between the non-spatial and spatial models may be easily understood and predicted, we emphasize their quantitative importance. If one were to fit a non-spatial telegraph model to nuclear molecular counts with some Fano factor to infer the mechanisms of transcriptional activity [73], the underlying dynamics may be incorrectly recovered and could lead to erroneous results. It is worth noting that transcriptomic measurements are more commonly total cellular counts, including the nucleus and cytoplasm. Assuming that degradation rates differ between these regions, the discussed spatial processes would still impact the Fano factor for the total count, but further investigation of this extended model is deferred to future work.

### 3.6. Two-dimensional cell with spatial heterogeneities

We make one last note about the applicability of findings towards more realistic set‐ups. Undoubtedly, mRNA transport in the nucleus is not one dimensional, nor a spatially homogeneous process. The nucleus is crowded with various factors that are known to modulate mRNA motion [23,50]. Moreover, the geometry of the nucleus itself plays a role in the export process because the mRNA must be transported to the boundary to be exported. In this last demonstration, we highlight the ability to handle these challenges within the currently presented framework.

**Figure 7.**
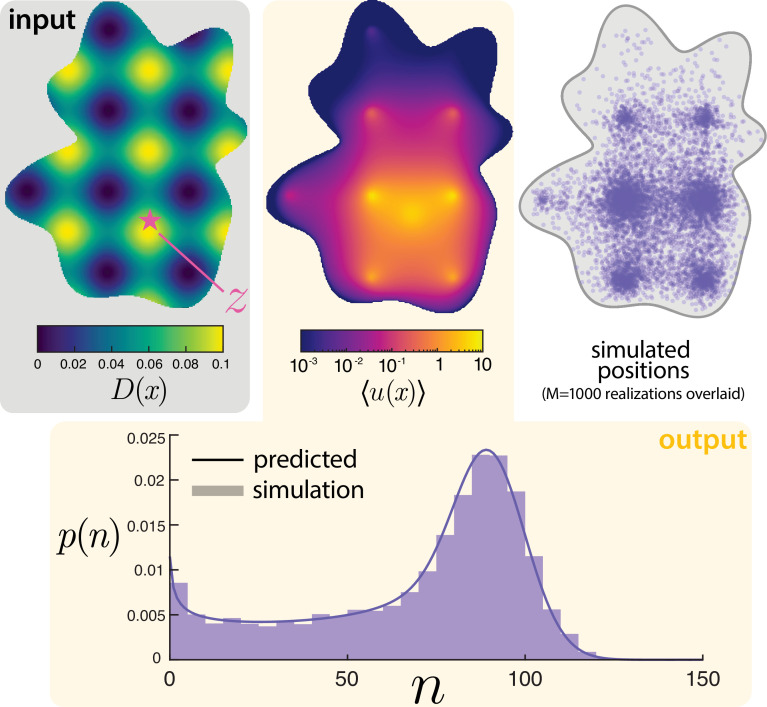
Generalizing the model to more realistic scenarios. The diffusivity D(x) is taken to be spatially varying (in the Ito sense) in a randomly generated two-dimensional domain. The mean number of particles ⟨u(x)⟩ is solved numerically from the PDE (3.18) and shown. The total mean is then used to specify the parameters of a Poisson-beta distribution for the total number of particles n, which shows close agreement with stochastic simulations.


In figure 7, we show a synthetically generated, two-dimensional cell geometry. The nuclear interior is assumed to be heterogeneous, modelled by a spatially dependent diffusion coefficient D(x). There are inherent technical challenges in interpreting spatially dependent diffusivities [74,75]. Here, we take an Itô interpretation with no justification beyond being the most straightforward to implement. In this vein, we also consider purely absorbing boundaries (κ=∞). How can the distribution of mRNA counts and positions in this spatially heterogeneous domain be predicted? Based on the findings thus far, both of these distributions can seemingly be accurately approximated by a single deterministic PDE solve for the mean intensity. Motivated by this observation, we use MATLAB’s PDE Toolbox to solve the deterministic equation

(3.18) $$\begin{array}{lcr} & \begin{array}{rl} 0 & =\nabla^{2}[D(x)\langle u(x)\rangle ]-\gamma \langle u\rangle +\lambda p\delta (x-z),\\ & \;\langle u(x)\rangle {|}_{x\in \partial \Omega }=0. \end{array} & \end{array}$$

The choice of Itô interpretation manifests in the location of the derivative with respect to the diffusivity. The solution of this PDE for ⟨u(x)⟩ is shown in figure 7 alongside M=1000 superimposed stochastic realizations of the particle positions. The prediction for positions and stochastic simulations demonstrate excellent agreement. Qualitatively, the molecules tend to get localized in regions of low diffusivity. Next, we turn to quantitative predictions. Motivated by the one-dimensional answers (equations (3.5) and (3.14)), we can define the analogous scale factor as


(3.19) $$\begin{array}{lcr} & S:=\frac{\int_{\Omega }\langle u(x)\rangle dx}{\lambda p/\gamma }. & \end{array}$$

This integral can be computed from the numerical PDE solution. Then, we posit that n approximately follows a Poisson-beta distribution with parameters $\tilde{\lambda }=S\lambda /\gamma$, $\tilde{\alpha }=S\alpha /\gamma$, and $\tilde{\beta }=S\beta /\gamma$. The resulting predicted distribution is shown in comparison against the counts from simulations in figure 7. This prediction using the numerical PDE solution for the scaling factor shows remarkable agreement with simulations. Although numerically solving a PDE on cellular geometries may be computationally costly, we emphasize that it seems far less costly than any current alternative approach for characterizing the distributions of both counts and positions. For instance, a generating function approach would seemingly require a PDE solution for each discretized pixel.

## 4. Inference on rates with heterogeneous cells

In this last section, we outline a possible avenue to use the findings in this work towards inference of model parameters with data. Although we have thus far achieved an understanding of the forward predictions of the model, the Cox process observations provide distinct technical challenges in their inference. The likelihood for a single observation (cell) of the Cox process (equation (2.1)) with parameters θ is [76]:


(4.1) $$\begin{array}{rl} L(\theta ;X) & :=p_{\theta }(X=x_{1},\ldots ,x_{n})=\int_{U}\left[\frac{1}{n!}e^{-\int_{\Omega }u(x)dx}\prod_{i=1}^{n}u(x_{i})\right]du(x). \end{array}$$

In other words, evaluation of the likelihood requires (infinite dimensional) integration over all possible realizations of the solution to the SPDE (2.2). While there is extensive literature on sophisticated numerical approaches for performing inference with this Cox process likelihood [76], we instead focus on the possibility of a simpler approximation that uses our earlier discussed findings.

As a motivating detour, we momentarily consider the likelihood of a simple Poisson process with deterministic intensity u(x) and underlying parameters θ. In this case, the likelihood becomes the term inside the integral of the Cox process likelihood:


(4.2) $$\begin{array}{lcr} & L_{\mathrm{Poiss}}(\theta ;X)=\frac{1}{n!}e^{-\int_{\Omega }u(x)dx}\prod_{i=1}^{n}u(x_{i}) & \end{array}$$

The observation we hope to emphasize can be seen by rearranging this likelihood and denoting $\langle n\rangle =\int_{\Omega }u(x)dx$ and $p(x_{i})=u(x_{i})/\langle n\rangle$. Then, the likelihood (equation (4.2)) can be written as


(4.3) $$\begin{array}{lcr} & \begin{array}{rl} L_{\mathrm{Poiss}}(\theta ;X) & =\frac{\langle n\rangle^{n}e^{-\langle n\rangle }}{n!}\prod_{i=1}^{n}p(x_{i})\\ & =f_{\mathrm{Poiss}}(n;\langle n\rangle )\prod_{i=1}^{n}p(x_{i}). \end{array} & \end{array}$$

In other words, for the Poisson spatial point process, the likelihood can be decomposed into the contributions of the counts and positions. Motivated by this observation and the findings presented thus far, we approximate the full Cox process likelihood by


(4.4) $$\begin{array}{rl} L(\theta ;X) & =\int_{U}\left[\frac{1}{n!}e^{-\int_{\Omega }u(x)dx}\prod_{i=1}^{n}u(x_{i})\right]du(x)\approx p_{\theta }(n)\prod_{i=1}^{n}p_{\theta }(x_{i}), \end{array}$$

where the spatial distribution is computed from the expected positions:


(4.5) $$\begin{array}{lcr} & p_{\theta }(x)=\frac{\left\langle u(x)\right\rangle }{\left\langle n\right\rangle }=\frac{\left\langle u(x)\right\rangle }{\int_{\Omega }\langle u(x)\rangle dx}. & \end{array}$$

Aside from the motivating example with deterministic intensity u(x), this approximation does not seem to be exact. Instead, equation (4.4) can be interpreted as a ‘mean-field’ approximation that does not fully account for correlations between counts and the spatial positions. Importantly, however, the quantities in this approximation are all tractable using the previously discussed results of this work. The mean behaviours used in equation (4.5) can be computed exactly. The count distributions $p_{\theta }(n)$ were found to be well approximated by a Poisson-beta distribution with parameters computed from ⟨n⟩. Therefore, the evaluation of this approximate likelihood reduces to straightforward analytical or numerical solutions to a single PDE for the mean intensity. In most scenarios, we can imagine, this is far less costly than any Monte Carlo sampling technique for evaluating the true Cox process likelihood.

**Figure 8.**
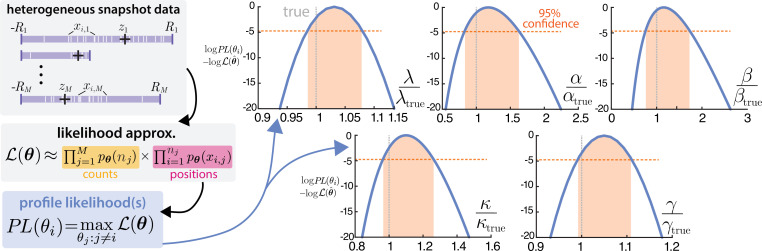
Demonstration of inference and identifiability on a heterogeneous dataset. A synthetic dataset of M=500 images, each with randomly chosen R and z was generated. This is then used in an approximation of the full likelihood (equation (4.4)) from which profile likelihoods are computed. The profile likelihoods are plotted as $\chi (\theta_{i}):=\log\;PL(\theta_{i})-\log\;L(\theta )$ to facilitate comparison with the threshold for an asymptotic confidence region for simultaneous inference of the parameters. Since all profile likelihoods decrease sufficiently fast over the windows of interest, structural and practical identifiability can be concluded.


The obvious question that remains is whether this approximation to the likelihood is sufficiently accurate to do reliable inference. We address this question with a demonstration of inference on synthetically generated data, presented in figure 8. To mimic the realistic challenge of cell-to-cell heterogeneity, we generate M=500 synthetic observations from the one-dimensional model, where each cell i has a randomly generated $z_{i}$ and $R_{i}$, but all kinetic parameters are fixed across the cells. We again assume steady-state conditions, so not all kinetic parameters are identifiable. We assume that the diffusion coefficient D may be measured through other means (for instance, live-cell tracking [50]), but the remaining parameters are unknown θ=[λ,γ,α,β,κ]. With this synthetic dataset, we use the Cox process likelihood approximation (equation (4.4)) for maximum likelihood inference using MATLAB’s fminsearch. To simultaneously diagnose the identifiability of these parameters, we also compute the profile likelihood for each parameter, defined by

(4.6) $$\begin{array}{lcr} & PL(\theta_{i}):=\max_{\theta_{j}:\;j\neq i}\;L(\theta ). & \end{array}$$

That is, the parameter of interest is fixed to a specified value (determined by the functional input to the profile likelihood) and the remaining parameters are optimized. The profile likelihood is a standard diagnostic for identifiability of parameters [77]. The output of each profile can be compared with the maximizing value of the likelihood over some window of interest, often corresponding to a confidence region. If the profile likelihood takes on sufficiently distinct values over this window, the parameter can be interpreted as both structurally and practically identifiable [78].

In figure 8, we see that the maximizing values of the profile likelihoods closely agree with the true simulated parameters. Moreover, we consider a window corresponding to the asymptotic 95% confidence region for the recovery of all parameters with a threshold of $\chi_{0.95,4}^{2}$, accounting for the four-degrees of freedom in the optimization of each profile likelihood [78]. Over this confidence region, all five parameters appear to be structurally and practically identifiable. We remind the reader of the non-spatial variant, a Poisson-beta distribution with only three identifiable parameters. It seems worth noting that all true parameters fall at the lower end of the confidence region, which may be indicative of biased estimates arising from this approximate inference procedure. We believe this proof-of-concept demonstration highlights how the findings of this work can be used for inference, but optimization and dissection of this inference are left for the future.

## 5. Discussion

We have formulated and investigated a toy model of nuclear mRNA that explicitly incorporates nuclear spatial diffusion and telegraph transcriptional dynamics. The most fundamental finding is that observations of the model form a Cox process, a spatial point process with intensity corresponding to the solution of a stochastically switching PDE. This spatial telegraph PDE lends itself to some analytical tractability in one spatial dimension. The mean and variance of both spatial distributions and counts were computed and verified against stochastic simulation. The model predictions about the role of spatial processes qualitatively agree with experimental findings, most notably that slowing down nuclear export increases the dispersion of nuclear mRNA counts [25]. However, these basic calculations were unwieldy even for the simplest of the models, making quantitative comparison challenging. The main upside of our work is the observation that a Poisson-beta distribution well approximates the full count distribution and parameters of this distribution can be straightforwardly computed. This distributional approximation was shown to generalize broadly, including Robin boundaries that model nuclear export and generically shaped two-dimensional cells with spatially heterogeneous diffusion. The computational tractability of the count distribution empowers the ability to perform approximate inference. We show that with heterogeneous snapshots of cells with distinct sizes and gene sites, kinetic parameters are identifiable from the spatial distributions and counts. Altogether, our work paves fundamental theoretical progress towards connecting imaging data (for instance, from smFISH) of spatial distributions of nuclear mRNA to infer the spatiotemporal gene expression dynamics underlying them.

Our findings should not be viewed as in tension with work that models gene expression dynamics non-spatially, for instance, those that treat mRNA export as a unimolecular reaction between homogeneous nuclear and cytoplasmic compartments [36,40]. Instead, our work sheds light on why these non-spatial models have found such success in their ability to fit observed RNA counts. Even with explicit spatial diffusion, nuclear export, and spatial heterogeneities, we found that a Poisson-beta distribution, the same prediction as the non-spatial process, well-describes the count distribution across parameters. The observation that explicit diffusion until export in the nucleus can be modelled as a single-step reaction has been noted before [25], but we emphasize that care must be taken in interpreting the parameters. For instance, a decay term must be interpreted as a mix of degradation and spatial export, and our findings show how these terms are differently affected by spatial geometric factors. It seems challenging, perhaps impossible, to disentangle these effects through fitting to non-spatial models. By contrast, we have shown that fitting the distributional counts with spatial information empowers a new resolution of detail to interrogate the dynamics.

On the biological relevance of the work, the model is a caricature of reality and should be treated as such. We have chosen various parameters throughout only to highlight qualitative features of the model. Although the nucleus is crowded and heterogeneous, approximately diffusive motion has been observed for mRNA [50] with a diffusion coefficient on the order of D≈0.03 [23] to D≈0.12 µm s^−1^ [79]. The Kuramoto length $\ell =\sqrt{D/\gamma }$ has been previously estimated to be µm for nuclear mRNA. The accordance of this length with nuclear diameters [29] suggests informational value in the spatial patterns. Moreover, these relative scales give a diffusive timescale of $R^{2}/D\approx 1$ h. This is in agreement with the observation that export and degradation are on approximately the same time scales [25]. However, nuclear export is not instantaneous, nor occurs at every location on the nuclear surface [24,27]. This suggests finite κ seems most appropriate, but we were unable to identify an approximate numerical value. It is unlikely that the full spatial distribution of diffusion coefficients can be identified as in figure 7. However, we believe this demonstrates the possibility of imaging nuclear condensates (nucleoli, speckles) that are known to mediate spatial organization of mRNA [23,80] and using this spatial information in the modelling and inference process. There is evidence nuclear mRNA degradation is far more elaborate than considered here [81], but an effective rate serves as a starting point for approximating these complexities. The transcriptionally active gene site was assumed to be fixed in location based on extensive experimental [42,82] and computational [83] evidence of chromosomal territories. This confinement leads to an effective diffusivity several orders of magnitude lower than that of mRNA. In the model, the point source could be replaced by a more realistic steady-state distribution reflecting this DNA diffusion with no apparent technical difficulties.

The telegraph process for transcriptional dynamics was chosen here as a minimally complex example of transcriptional dynamics that produces super-Poissonian dispersion of molecular counts [84]. This dispersion is intimately linked with the correlation between the particles induced by the production process and causes the particle-wise machinery of our previous work [49] to fail. We anticipate this approach lends itself better to generalizing into other complexities of interest. In this vein, there are several avenues of promising future direction. We have considered only a single mRNA population, but one could imagine extending the framework to account for multiple species available from imaging such as multiple genes [31,85] or both nuclear and cytoplasmic RNA [31,40]. The telegraph transcriptional process also lends itself to generalization to more realistic multi-state production processes [86–90], feedback (with delay) [11,91], or time-dependent cell-cycle dynamics [13,63,92]. Even more broadly, we hope the machinery of this work can be used to study other spatial regulations of gene expression [93,94] that occur with distinct subcellular localization such as RNA splicing [29,95].

On the theory and computational side, the finding of a Poisson-beta distribution for the counts was heuristically motivated. Owing to the lack of proof, it remains unclear whether the distribution of counts genuinely follows this distribution or is merely well approximated by it. Over a broad set of parameters, the approximation did not exhibit any discernible pattern of failure. We believed that the Poisson-beta approximation corresponds to a truncation of the Green’s function series. However, the leading term of the series solution (equation (3.7)) does not seem to follow the form (equation (3.10)) conjectured from scaling arguments. Nonetheless, we believe this line of investigation is worthwhile to share and warrants further investigation in the future owing to its computational tractability. Alternative computational approaches that seem likely to be fruitful include simulation-based inference [96,97] and graph neural networks for stochastic reaction-diffusion that can generalize to arbitrary geometries [98].

## Data Availability

MATLAB code to generate all stochastic simulations and analysis thereof has been archived on Zenodo and can be found at [99].

## Appendix A. Poisson representation of the model

In this section, we provide further details on the claim in the main text that the observations of the discrete particle process follow a Cox process (equation (2.1)) with intensity governed by the SPDE (2.2). The idea of spatial Poisson representations dates back to Gardiner [56,57]. More accessible recent work relating mechanistic models and point processes can be found in [48,100] and the geophysics literature [64]. We believe the explicit computation of this model with dichotomous noise has not been considered.

For simplicity that can be easily generalized, consider a one-dimensional domain discretized into windows of size Δx, each with counts $n_{1},\ldots ,n_{M}$. Denote the promoter state corresponding to Λ(t) in the main text as j(t)={0,1}. In state j=0, transcription occurs at rate ${\tilde{\lambda }}_{\mathrm{off}}=\lambda_{\mathrm{off}}(\Delta x{)}^{-1}$ and in state j=1, at rate $\tilde{\lambda }=\lambda (\Delta x{)}^{-1}$. The gene site is fixed to be in the spatial location indexed k. In the discretized model, diffusion corresponds to a unimolecular reaction at rate $D=D/(\Delta x^{2})$.

Denote $p_{0}(n,t)=p(n,0,t)$ and $p_{1}(n,t)=p(n,1,t)$, respectively. The stochastic vector of counts n and promoter state j evolves through the Chapman–Kolmogorov equations:

(A 1)
$$\begin{array}{rl} \frac{\partial p_{j}}{\partial t}(n,t)= & \sum_{i=1}^{n}D(n_{i+1}+1)p_{j}(n+r_{i+1}^{i})+D(n_{i-1}+1)p_{j}(n+r_{i-1}^{i})-2Dn_{i}p_{j}(n,t)+\gamma (n_{i}+1)p_{j}(n+r_{i}^{+},t)-\gamma n_{i}p_{j}(n,t)\\ & +{\tilde{\lambda }}_{off}p_{j}(n+r_{i}^{-},t)\delta_{j0}\delta_{ik}-{\tilde{\lambda }}_{off}p_{j}(n,t)\delta_{j0}\delta_{ik}+\tilde{\lambda }p_{j}(n+r_{i}^{-},t)\delta_{j1}\delta_{ik}-\tilde{\lambda }p_{j}(n,t)\delta_{j1}\delta_{ik}. \end{array}$$

We have abbreviated the perturbation vectors $r_{i}^{\pm }=\left[0,\ldots ,\pm 1,0,\ldots \right]$ and $r_{i}^{i+1}=\left[0,\ldots ,-1,+1,0,\ldots \right]$. The Poisson representation takes the ansatz:

(A 2)
$$\begin{array}{lcr} & p_{j}(n,t)=\int_{u_{i}}\prod_{i=1}^{M}\frac{e^{-u_{i}}u_{i}^{n_{i}}}{n_{i}!}f_{j}(u,t)du_{i}, & \end{array}$$

which gives the evolution of the intensities as

(A 3)
$$\begin{array}{rl} \frac{\partial f_{j}}{\partial t} & =-\sum_{i=1}^{M}\frac{\partial }{\partial u_{i}}\left[(Du_{i+1}+Du_{i-1}-2Du_{i}-\gamma u_{i}+{\tilde{\lambda }}_{off}\delta_{j0}\delta_{ik}+\tilde{\lambda }\delta_{j1}\delta_{ik})f_{j}(u,t)\right]+\sum_{q=0,1}A_{jq}f_{q}(u,t), \end{array}$$

where A is the 2×2 generator matrix for the promoter switching process. This is the Chapman–Kolmogorov equation for the piecewise deterministic Markov process (PDMP) system:

(A4)
$$\begin{array}{r} \begin{array}{rl} \frac{du_{i}}{dt} & =Du_{i+1}+Du_{i-1}-2Du_{i}-\gamma u_{i}+{\tilde{\lambda }}_{off}\delta_{ik}\\ & \beta ↑↓\alpha \\ \frac{du_{i}}{dt} & =Du_{i+1}+Du_{i-1}-2Du_{i}-\gamma u_{i}+\tilde{\lambda }\delta_{ik}. \end{array} \end{array}$$

In the limit as Δx→0 with $u(x,t)=u_{i}(t)/\Delta x$, the switching PDE (2.2) is recovered. We note the structural contrast of this result compared with related works [48,64] that arrive at Gaussian SPDEs from biomolecular reactions or white-noise limits.

## Appendix B. Stochastic simulation details

For all stochastic simulations, the promoter state switching, birth of molecules, and their decay are handled by a standard Gillespie simulation [43] until some time T. To ensure steady state, T is chosen to be $T=100/\;\min\{\alpha ,\beta ,\gamma ,\lambda ,R^{2}/D\}$. For all particles living at the end of the simulation, their birth times are recorded. These candidate molecules are then simulated spatially, starting at their birth time until either T or they exit the domain. The final positions of all remaining molecules that have survived the Gillespie and spatial simulation steps are outputs of the simulation. In simulations with finite κ, the boundary is assumed to be a partially reflected diffusion [72]. If a particle Euler steps out of the domain, with probability PΔt it is absorbed. Based on that work, $P=\kappa \sqrt{\pi /D}$. If the particle is not absorbed, it is then reflected. All finite κ simulations throughout the article are in one spatial dimension, where reflection is straightforward. In simulations with heterogeneous D(x), the Euler step is interpreted in an Itô sense ,$x(t+\Delta t)=x(t)+\xi \sqrt{2D(x)\Delta t}$, ξ∼N(0,1). [75].

### (a) Parameter values used in figures

Figure 2: z=0, R=3, α=0.5, β=0.1, λ=100, $\lambda_{\mathrm{off}}=0$, γ=1.5, D=1. $N_{\mathrm{sims}}=5000$.

Figure 3: z=0, R=3, α=0.5, β=0.1, λ=100, $\lambda_{\mathrm{off}}=0$, γ=1.5, D=1. $N_{\mathrm{sims}}=5000$.

Figure 4: z=0, R=3, α=0.5, β=0.1, λ=100, $\lambda_{\mathrm{off}}=0$, γ=1.5, D=1. $N_{\mathrm{sims}}=5000$.

Figure 5: z=0, R=3, α=0.5, β=0.1, λ=100, $\lambda_{\mathrm{off}}=0$, γ=1.5, D=5. $N_{\mathrm{sims}}=5000$.

Figure 6: z=0, R=1, α=0.5, β=0.1, λ=100, $\lambda_{\mathrm{off}}=0$, γ=0.75, D=1, panels b–d: $\kappa =10^{3}$.

Figure 7: z=[0,−0.5], ⟨R⟩=1, α=0.75, β=0.25, λ=100, $\lambda_{\mathrm{off}}=0$, γ=1, $D(x,y)=D_{-}+(D_{+}-D_{-})(\cos(3\pi x)+\cos(3\pi y)+2)/4,$, $D_{+}=0.1$, $D_{-}=10^{-3}$, $N_{\mathrm{sims}}=5000$.

Figure 8: domains are randomly generated by $R_{i}∼\Gamma (4,1/4)$, $z_{i}∼\beta (4/3,4/3)\times 2R-R$, so ⟨R⟩=1,var⁡(R)=0.5, ⟨z⟩=0,var⁡(z)≈0.34. The remaining parameters are defined the same as the main text and taken to be: α=1, β=0.25, λ=250, $\lambda_{\mathrm{off}}=0$, γ=1.5, D=1, κ=5, $M_{\mathrm{data}}=500$.

## Appendix C. Series solution for variance of population

In this section, we show the derivation of the variance result in §3.2. The calculations closely follow [67] on the stochastic cable equation. As noted in equation (3.8), the SPDE (2.2) can formally be solved by integrating the stochastic point source noise against the Green’s function G(x,t) that satisfies

(C 1)
$$\begin{array}{lcr} & \begin{array}{rl} & \partial_{t}G=D\partial_{xx}G-\gamma G,\\ & G(-R,t)=0,\;G(R,t)=0,\\ & G(x,0)=\delta (x-z). \end{array} & \end{array}$$

This Green’s function is [69]:

(C 2)
$$G(x,t)=\sum_{m=1}^{\infty }g_{m}(t)\phi_{m}(x)\phi_{m}(z),$$

with

(C 3)
$$\begin{array}{lcr} & \begin{array}{rl} g_{m}(t) & :=e^{-t(\gamma +D\pi^{2}m/(2R{)}^{2})}:=e^{-\mu_{m}t}\\ \phi_{m}(x) & :=\sqrt{1/R}\sin(m\pi (x+R)/2R). \end{array} & \end{array}$$

By equation (3.6), computation of the variance of n requires knowledge of the second moment of the underlying stochastic intensity. That is:

(C 4)
$$\begin{array}{rl} C(x,y,t) & =\langle u(x,t)u(y,t)\rangle \\ & =\langle \left[\int_{0}^{t}G(x,t-s_{1})\Lambda (s_{1})ds_{1}\right]\left[\int_{0}^{t}G(x,t-s_{2})\Lambda (s_{2})ds_{2}\right]\rangle ds_{2}ds_{1}\\ & =\int_{0}^{t}\int_{0}^{t}G(x,t-s_{1})G(y,t-s_{2})\langle \Lambda (s_{1})\Lambda (s_{2})\rangle ds_{2}ds_{1}. \end{array}$$

The autocorrelation of the dichotomous process is classical [62], and satisfies

(C 5)
$$\begin{array}{lcr} & \langle \Lambda (s_{1})\Lambda (s_{2})\rangle =\frac{\lambda^{2}\alpha \beta }{(\alpha +\beta {)}^{2}}e^{-|s_{1}-s_{2}|(\alpha +\beta )}:=\kappa e^{-|s_{1}-s_{2}|\omega }. & \end{array}$$

Ultimately, this gives us

(C 6)
$$\begin{array}{rl} C(x,y,t) & =\kappa \sum_{n=1}^{\infty }\sum_{m=1}^{\infty }\phi_{n}(x)\phi_{n}(z)\phi_{m}(y)\phi_{m}(z)\int_{0}^{t}\int_{0}^{t}g_{n}(t-s_{1})g_{m}(t-s_{2})e^{-|s_{1}-s_{2}|/\tau }ds_{2}ds_{1}. \end{array}$$

We defer evaluating this, noting that ultimately we want to spatially integrate C(x,y,t), and that

(C 7)
$$\begin{array}{rl} & \int_{-R}^{R}\int_{-R}^{R}\phi_{n}(x)\phi_{m}(y)\phi_{n}(z)\phi_{m}(z)dxdy=\frac{4\left((-1{)}^{m}-1\right)\left((-1{)}^{n}-1\right)\phi_{m}(z)\phi_{n}(z)}{\pi^{2}mn}. \end{array}$$

With some help from Mathematica, we can also integrate

(C 8)
$$\begin{array}{lcr} & \tilde{g}(t):=\int_{0}^{t}\int_{0}^{t}g_{m}(t-s_{1})g_{m}(t-s_{2})e^{-|s_{1}-s_{2}|\omega }ds_{2}ds_{1}. & \end{array}$$

The expression is unwieldy, but after taking $\lim_{t\to \infty }\;$, we get

(C 9)
$$\begin{array}{rl} \tilde{g} & =\lim_{t\to \infty }\tilde{g}(t)=\frac{\mu_{m}+\mu_{n}+2\omega }{\left(\mu_{m}+\omega )(\mu_{n}+\omega )(\mu_{m}+\mu_{n}\right)}. \end{array}$$

The temporal terms (equation (C 9)) and spatial terms (equation (C 7)) can be combined into equation (C6) to nearly give the infinite sum in the main text. The last ingredient is the observation that only odd terms of (equation (C 7)) are non-zero, so we re-index m→2m−1, n→2n−1, and equation (3.7) is recovered. Figures with predictions from the series truncate after 1000 terms.
